# How to Use Artificial Intelligence to Improve Entrepreneurial Attitude in Business Simulation Games: Implications From a Quasi-Experiment

**DOI:** 10.3389/fpsyg.2022.856085

**Published:** 2022-04-13

**Authors:** Jiachun Chen, Yuxuan Chen, Ruiqiu Ou, Jingan Wang, Quan Chen

**Affiliations:** ^1^Department of Management, Zhongshan Institute, University of Electronic Science and Technology of China, Zhongshan, China; ^2^School of Economics and Management, Hanshan Normal University, Chaozhou, China

**Keywords:** business simulation game, artificial intelligence, entrepreneurial attitude, quasi-experiment, fuzzy set qualitative comparative analysis, process perspective

## Abstract

Business simulation games (BSGs) have been widely used in entrepreneurship education with positive effects. However, there are still some deficiencies in the BSGs, such as limited guidance, low uncertainty and limited simulation environment, which make it impossible to exert the maximum effect. Artificial intelligence (AI) can solve the above shortcomings. The combination of AI and BSGs is the possible development direction of BSGs. But how to effectively combine BSGs with AI is still an open question. Using a quasi-experimental design, this study uses fuzzy-set qualitative comparative analysis to analyze how participants’ entrepreneurial attitude changes in BSGs. The results show that BSGs can effectively improve entrepreneurial attitude, and there are four types of promotion configurations. These four configurations consist of five antecedent conditions. According to the above conclusions, AI can improve entrepreneurial attitude in BSGs in various ways, such as simulating competitors, providing targeted feedback for failures, and improving game experience. The contribution of this paper is to highlight the possibility of combining AI with BSGs, and to provide suggestions on how AI can intervene in BSGs.

## Introduction

COVID-19 pandemic imposes restrictions on social distancing, enabling mobile learning to be widely adopted in universities (Yuan et al., 2021; Zea et al., 2021). Distance and digital learning have also been rapidly promoted in entrepreneurship education (Zulfiqar et al., 2021), because it is more adaptable to the development of the times and promotes students to obtain educational resources as much as possible (Ferreira et al., 2021). As a virtual dynamic learning tool, business simulation games (BSGs) have been developed as a realistic learning strategy, which are currently recognized by more and more scholars in entrepreneurship education (Faria et al., 2009). As one of the experiential learning tools (Hägg and Gabrielsson, 2019), BSGs are regarded as a model of practical learning in the entrepreneurial field. Academia and business are increasingly using BSGs to encourage participants in decision-making, risk management, communication and teamwork (Costin et al., 2018). In addition, BSGs have the advantages of low cost, low risk, and repeatability (Fox et al., 2018), which is not limited to specific locations (Zulfiqar et al., 2021). The possible value of BSGs to education is also a focus of future educational research (Bondar et al., 2020; Zulfiqar et al., 2021).

However, BSGs have some inherent shortcomings, which make their effects in entrepreneurship education not maximized, and the intervention of artificial intelligence (AI) can solve these problems. The development of new technologies such as mobile technology (Chen et al., 2015, 2019a,^2022^), cloud computing (Huang et al., 2012, 2019; Chen et al., 2019b; Lin et al., 2020), and AI technology has made it possible to improve business simulation games (Liu et al., 2014; Huang et al., 2015, 2017). The application of AI in games can deal with some challenging problems in virtual or real environments (Salta et al., 2021). First, a disadvantage of BSGs is the low level of uncertainty (Politis and Gabrielsson, 2009). It is very necessary to introduce the concept of uncertainty in BSGs. Entrepreneurs often need to make decisions in the face of an uncertain environment, but BSGs simulate a relatively stable environment with a low degree of uncertainty. And uncertainty is a difficult concept to model (Fox et al., 2018). The intervention of AI makes it possible for the diverse contexts of BSGs, and contextual learning is an important consideration for game designers in entrepreneurship education (Zulfiqar et al., 2019). On the one hand, AI can also provide different simulation scenarios for participants with different course requirements and ability levels by changing different parameter configurations (Bikovska et al., 2006). This satisfies the participant’s requirement for individuality and adaptability of the simulated game process (Kuleto et al., 2021). On the other hand, the use of AI technology can effectively avoid human factors of unreliability and instability, thus improving the quality of simulation games (Bikovska et al., 2006). Secondly, the role of teachers in BSGs is limited. It is important for teachers to provide timely feedback to students in BSGs (Thanasi-Boçe, 2020). However, the ability of teachers is limited. In BSGs, multiple teams need to make decisions in a short period of time to fight against each other. It is impossible for teachers to analyze the business results of each team separately so as to provide effective feedback. AI can partially replace teachers’ decision-making, make strategic or tactical decisions (Shim et al., 2002), and provide timely feedback. AI can provide specific tasks and intelligent decision support through automated data integration, improving clarity and simplicity for participants (Brauner et al., 2019). According to existing research, the degree of reference of participants to AI decision-making is proportional to the complexity of the task (Brauner et al., 2019), can improve the efficiency of decision-making (Röttger et al., 2009). At the same time, AI can also determine the level of support and automation according to the different cognitive and skill levels of participants (Brauner et al., 2019). Thirdly, the immersive experience in BSGs is very important (Newbery et al., 2018). If AI can investigate and record the behaviors and reactions of participants, and determine which aspects of the game experience are good, it can provide suggestions for improving the design of the game. AI technology can provide participants with an engaging, challenging and fun gaming environment (Dreżewski and Solawa, 2021), which is conducive to a deep learning experience, thereby promoting an immersive experience. Students learn better when they construct knowledge in a more engaging environment (Lopez-Gazpio, 2022). AI can also collect participants’ motivation and attention levels in different links through neuroscience techniques such as electroencephalogram signal and eye tracking, so as to fully understand the participants’ gaming experience and the attractiveness of the game (Ferreira et al., 2021). Finally, AI can save and share game data, which can help reduce the time and cost of the game (Matulis and Harvey, 2021), facilitate quick access to educational support for students (Kuleto et al., 2021),thereby promoting the maximum effect of entrepreneurship education. The more different situations an AI accumulates, the more conducive it is to function (Bikovska et al., 2006). AI can also automatically correct mistakes and omissions in the game process based on participants’ game data to continuously develop and improve game modes (Xi, 2020). At the same time, because it involves the monitoring of the entire game process, it is difficult to evaluate the real-time evaluation of the realization process of the game goal and the actual performance of the participants (Ferreira et al., 2021), which is also a problem that AI can help to solve.

Business simulation games use “simulation” to promote students to improve their entrepreneurial practice ability in scenario learning. The design of the BSGs is to construct a real business environment and show the overall situation of the company’s strategic functions in a virtual environment, prompting students to improve their entrepreneurial knowledge and skills from the process of managing a company in a risk-free environment (Costin et al., 2018). It helps students understand the relationship between different processes and businesses, and understand the consequences of some unscientific decisions, such as performance-oriented decisions (Barnaby et al., 2021). BSGs assume different scenarios connected to the real business world (Zulfiqar et al., 2021), prompting participants to combine learning theory with entrepreneurial practice to improve entrepreneurial skills. BSGs place students in a fierce market competition environment through simulation, and encourage them to avoid risks by analyzing the situation of the market and competitors (Thanasi-Boçe, 2020). Simulation can reflect the imperfect problems in the real market environment. The limited time and resource pressure set by the simulation will encourage students to improve the efficiency of teamwork and the ability to make judgments (Thanasi-Boçe, 2020). In order to surpass rivals in the fierce market competition, simulation requires participants to make reasonable resource allocation decisions and smoothly realize product production and sales, which can effectively enhance students’ entrepreneurial awareness and enhance entrepreneurial skills (Fox et al., 2018). Therefore, the authenticity, repetitive operation, and result monitoring functions of the simulation process help participants better familiarize themselves with the rules of the real business world (Hsieh et al., 2016) and enhance their entrepreneurial practice capabilities.

Entrepreneurship attitude is an important indicator of the expression of entrepreneurial emotions. Nabi et al. (2017) found that most studies use entrepreneurial intentions to measure the effect of entrepreneurship education, and need to consider emotional expression factors such as student feelings and attitudes. The higher the entrepreneurial attitude, the easier it is for individuals to become entrepreneurs (Kolvereid, 1996). Therefore, it is necessary to deeply study the connotation, influence mechanism and change process of entrepreneurial attitude, which will help to better carry out entrepreneurship education and cultivate entrepreneurial talents. Entrepreneurship education is an external factor that leads to the change of attitude and intention (Souitaris et al., 2007). Studies have shown that entrepreneurial attitude, as one of the motivational antecedents of entrepreneurial intention, has an important impact on entrepreneurial intention (Krueger et al., 2000; Liñán and Chen, 2009). Practice is the main reason for attitude changes, which in turn affect behavior changes. Entrepreneurship attitude is the emotional expression of becoming an entrepreneur, and it is the first question that entrepreneurs think about when they really start entrepreneurial behavior (Souitaris et al., 2007). Many scholars mentioned that entrepreneurship education should pay attention to the improvement of all participating students’ awareness of entrepreneurship (Jones, 2010) and changes in entrepreneurial mentality (Daniel, 2016). Therefore, improving students’ entrepreneurial attitude is an important goal of entrepreneurship education.

At present, relevant research rarely combines the improvement of entrepreneurial attitude with the process of BSGs. This is not conducive to studying how BSGs change entrepreneurial attitude from a process perspective. Moreover, research methods such as longitudinal tracking in related studies are still rare. How the process of BSGs affects entrepreneurial attitude remains less explored. More importantly, there is still a lack of research on the development process of specific entrepreneurial teaching methods (Souitaris et al., 2007), especially the research directly linking the results of entrepreneurial education with entrepreneurial teaching methods (Pittaway and Cope, 2007). According to existing research, the role of teachers, students’ participation enthusiasm, failure in the game process (Hou, 2015), the performance of simulation companies (Thanasi-Boçe, 2020) and the flow experience of students (Hou and Li, 2014; Yen and Lin, 2020) are important research variables in BSGs. The emotions, methods and results in the process of participating in BSGs will affect the students’ entrepreneurial attitude and other entrepreneurial education results.

In order to explore the impact of BSGs on entrepreneurial attitude and how the game process affects entrepreneurial attitude, this paper adopts experimental methods, questionnaires and qualitative comparative analysis. Experimental methods are widely used in educational research. Experiments expose students of the same background to different teaching methods and compare specific research variables (such as attitudes and learning behaviors). The ideal result of the experimental method is to determine the causal relationship behind the relevant variables through the comparison of the experimental group and the control group (Charness et al., 2012). In addition, different data will be obtained between variables and different combinations of variables under the experimental method (Hsee and Leclerc, 1998). Therefore, the experimental method can extract the research variables in the process of BSGs, and explore the correlation between different variables and their combinations that affect entrepreneurial attitude. Through the comparison between the experimental group and the control group, we can deeply analyze the promotion mode of entrepreneurial attitude in different situations, and draw research conclusions more objectively. Questionnaires were used in the experiment to measure the relevant variables before and after the experiment. The goal of using the qualitative comparison method (QCA) is to explain in more detail the impact of BSGs on college students’ entrepreneurial attitude. The qualitative comparison method (QCA) is suitable for analyzing a variety of different situations (Ragin, 2010), and can deeply explore the reasons and effects of different combinations of situations. Among them, Kraus et al. (2018) mentioned that the use of fsQCA in articles on entrepreneurship and innovation research is increasing, and the application of fsQCA has unlimited potential in the future. In recent years, the many advantages of fsQCA have also led to its increasing application in social science research (Jiang et al., 2021). FsQCA is especially good at causal relationship analysis, which can further reveal complex relationships between variables (Yu et al., 2021). At the same time, fsQCA does not affect the overall analysis due to changes in variables, but only affects the combination of variables (Jiang et al., 2021), which is more scientific than traditional analysis methods (Liu et al., 2017). Therefore, it is suitable for our analysis of the influence of different variables and their combinations involved in the development of BSGs on entrepreneurial attitude.

## Literature Review

### Business Simulation Game

The purpose of BSGs is to develop practical entrepreneurial skills of participants such as analysis, decision-making, organization and management by understanding the process of establishing a company (Zulfiqar et al., 2021). Participants continuously evaluate and improve group strategic, financial, human resources and other management decisions in order to apply the acquired knowledge and skills to future entrepreneurial practice (Costin et al., 2018). BSGs reflect the strategic situation of the enterprise by simulating the operation of the enterprise and the trend of the market (Toma et al., 2020).

As a game, reflecting the attributes of the game and the characteristics of game participation is another important goal of BSGs. Game attributes such as goals, rules, levels, status, competition, results, leaderboards, rewards, etc., are the attributes of the simulation game (Fox et al., 2018), which can effectively encourage participants to learn new knowledge and take new actions. Interaction, entertainment, infectiousness, ease of learning, perceived usefulness, knowledge sharing, and knowledge applicability are the characteristics of game participation, which can encourage students to be more interested in participating in it and help them think about their future career directions (Zulfiqar et al., 2021). Fun and enjoyment, as the basic characteristics of the game, have a positive relationship with students’ interest and participation, which is conducive to improving students’ decision-making confidence and motivating students to achieve their goals (Mardani et al., 2018). It is worth mentioning that the entertainment in BSGs is not simple sensory entertainment, but with education as the ultimate goal (Pando-Garcia et al., 2016). Studies have shown that using game attributes to understand whether students have a satisfactory learning experience and pleasant evaluation during the participation process can better enhance their attitudes toward entrepreneurship (Ranchhod et al., 2014). In addition, the relatively closed and controlled atmosphere provided by BSGs also helps participants to have a strong sense of self-efficacy in them (Dumblekar and Dhar, 2021).

### Business Simulation Games in Entrepreneurship Education

The entrepreneurial ability of students can be obtained in entrepreneurial education. The essence of entrepreneurship education is to gain entrepreneurial experience and problem-solving methods by participating in real-world practice (Zulfiqar et al., 2021). Basic knowledge in entrepreneurship education can be achieved through traditional classroom teaching, but the learning of entrepreneurial skills and mentality requires innovative methods, such as BSGs (Zulfiqar et al., 2021). It has been more than 50 years since BSGs were set up as a university course (Goi, 2019). Meanwhile, BSGs are widely used methods in entrepreneurship education (Fox et al., 2018). For example, 1,700 colleges and universities in the United States use BSGs to improve students’ entrepreneurial skills and entrepreneurial intentions (Zulfiqar et al., 2019). Germany also attaches great importance to BSGs in the field of entrepreneurship education (Kriz and Auchter, 2016). At present, the exploration and application of BSGs in entrepreneurship education still needs to be developed (Ratten and Jones, 2021).

From the perspective of teacher teaching, many scholars believe that entrepreneurship teachers should innovate teaching methods and use simulation games in entrepreneurship education courses to truly develop students’ entrepreneurial abilities (Faria et al., 2009; Wood et al., 2009) and overall quality (Isabelle, 2020). Using BSGs as the first link in the teaching of entrepreneurship education is conducive to better guidance and assistance for teachers in subsequent teaching (Pérez-Pérez et al., 2021). The application of BSGs for teaching is an important manifestation of teachers’ use of action pedagogy. By involving students in the daily work of entrepreneurs of BSGs (Lightner et al., 2007; Page and Mukherjee, 2007), it promotes in-depth learning. Experiential learning enables better understand entrepreneurial practice and entrepreneurship (Tenenbaum et al., 2001). Immediate feedback is an important manifestation of simulation games as a reliable teaching tool, which helps reduce students’ uncertainty about the market environment (Oblinger, 2014). As a result, BSGs also put forward higher requirements for the teacher’s “guidance” role in teaching, rather than the traditional “authoritative” teaching. In addition, some scholars have pointed out that BSGs can be used as an emerging teaching method that can serve as a reference for other disciplines beyond entrepreneurship courses (Costin et al., 2018). From the perspective of students’ learning subjects, BSGs are the practice of constructivist learning theory, because they can help students construct new knowledge and better reserve knowledge in simulated learning, which can reduce the pressure of learning (Zulfiqar et al., 2021). As a tool for students’ active learning, BSGs reflect the characteristics of demonstration, science and practice, which promote the development of knowledge and skills (Ferreira et al., 2021). The requirement of teamwork in BSGs also helps students to form a good and independent learning character (Buzady and Almeida, 2019).

### Artificial Intelligence and Business Simulation Games

The current research on AI in BSGs is still relatively rare, but it is necessary to add AI to BSGs (Fuchsberger, 2016). As a new technology with high technology, interdisciplinary (Kuleto et al., 2021), wide application (Xi, 2020), strong adaptability and stability (Yang et al., 2021), its participation can promote development of learning styles in simulation games (Fuchsberger, 2016). AI is widely used in simulation environments (Rao et al., 2011; Lam et al., 2012), especially to play a guiding role in the simulation teaching of education, and has gradually developed in the direction of intelligent expert systems (Fuchsberger, 2016). Due to the rapid development of the era of big data, it is difficult for the education system to quickly obtain the latest methods and adapt to the latest needs within a certain period of time (Ifenthaler and Egloffstein, 2020). The development of related algorithms has made the application of AI more extensive (Ye et al., 2019, Ye et al., 2021; Fu et al., 2022). AI helps greatly enrich and expand human learning, knowledge, and horizons through technologies such as perception, practice, imitation, integration, and sharing (Yang et al., 2021). The future development of AI is also closely related to human communication and cooperation (Yang et al., 2021), especially in the application of higher education with unlimited development potential (Kuleto et al., 2021). Many higher education institutions have applied artificial intelligence to teaching, providing teachers with more teaching support and better promoting students’ academic achievement (Salta et al., 2021).

Artificial intelligence in games has attracted a lot of attention and learning provides an environment for AI to be applied in various fields of learning (Agung and Gaol, 2012). Integrating AI technology with the game industry will help enrich the content and form of games, meet players’ higher pursuits and expectations for games, which promote the transformation and development of games (Xi, 2020). AI can also monitor the attention level and other mental states of participants throughout the process to understand the attractiveness of different game sessions, thereby optimizing the learning experience (Ferreira et al., 2021). According to research, in participating in educational training and learning, contemporary young students are accustomed to and like to use AI technology (Salta et al., 2021). AI has begun to show its advantages in gaming. Take the AI AlphaGo as an example, in the classic game of Go, the AlphaGo learns through various algorithms and beats the human professional players (Silver et al., 2016). AI can also compete as a player’s competitor in electronic competitions for driving simulated cars (Salta et al., 2021).

The application of AI in games is reflected in the functions of changing, creating different roles, scenarios for participants (Salta et al., 2021). It provides decision support to achieve real-time competition, challenge and serve players, which promote players to develop skills and other purposes (Salta et al., 2021). The application of AI may include the following aspects: firstly, AI in BSGs is served as simulate tutors or virtual players (Bikovska et al., 2006), and AI simulates decision-making and models expert knowledge. Secondly, AI generates simulated scenarios based on initial conditions that define the game or rules set by the game leader. Thirdly, AI can provide support for the decision-making of participants. With the emergence of AI, intelligent decision-making support systems has become a trend (Brauner et al., 2019).

From the current research, the discussion of how AI can be used in BSGs is still insufficient. Therefore, it is necessary to analyze the operation process of BSGs to understand which parts are conducive to promoting entrepreneurial learning, so as to clarify the possible application of AI in BSGs.

### Business Simulation Games and Entrepreneurial Attitude

Individual entrepreneurial attitude refers to the individual’s preference for engaging in entrepreneurial activities or participating in organizational work (Kolvereid, 1996), and also includes the degree of evaluation of the emotional preferences and practical benefits of becoming an entrepreneur (Autio et al., 2001). Attitude refers to a person’s emotional expression of a certain thing or situation, including positive and negative attitude types (Zimmerman, 2008). Attitude triggers intention, which leads to behavior (Ajzen, 1991). The importance of entrepreneurial attitude is that for students, the improvement of entrepreneurial attitude represents to a certain extent that students have a certain understanding of entrepreneurship and a positive attitude toward entrepreneurship. Many scholars also believe that the definition of entrepreneurship education should be broader, focusing on enhancing all students’ understanding of entrepreneurship after participating in entrepreneurship education, not limited to the career that students choose to pursue in the future (Jones, 2010). Therefore, the entrepreneurial attitude is a particularly important research object for the development of entrepreneurial education.

Business simulation games encourage students to form an entrepreneurial attitude (Donckels, 1991). Some scholars mentioned that in an academic environment, virtual games are used to study entrepreneurial attitude and the impact on entrepreneurship (Curry and Moutinho, 1992). Existing studies have shown that students’ feeling of benefit and happiness in participating in BSGs is beneficial to their entrepreneurial attitude (Zulfiqar et al., 2019), which encourages them actively participate in the learning of simulation games to enhance the understanding of entrepreneurial knowledge (Liu and Huang, 2015). Participating in BSGs will also make students feel the difficulty of becoming an entrepreneur, which is in line with the attitude of many entrepreneurs toward launching entrepreneurial activities in reality (Pérez-Pérez et al., 2021).

The characteristics and game attributes of BSGs are conducive to improving students’ entrepreneurial attitude. The three important characteristics of BSGs are simulation, verification, and effectiveness (Fox et al., 2018). These three aspects need to be implemented in the game process to allow students to have a sense of reality in running a business, which is beneficial to the improvement of entrepreneurial attitude and entrepreneurial intention. BSGs encourage students to think autonomously (Thanasi-Boçe, 2020), think creatively, and actively communicate and solve problems (Zulfiqar et al., 2021). Simulation promotes students to learn in action. While giving students the identity of entrepreneurs, role play in simulation also cultivate their passionate entrepreneurial attitude and practical entrepreneurial skills (Costin et al., 2018). Competitive factors in the simulation will increase students’ enthusiasm for participation, thereby enhancing entrepreneurial attitude. At the same time, the game attributes of BSGs, such as interactive, entertaining, infectious, and fun (Zulfiqar et al., 2021), not only immerse students in the process of enjoying the game (Ferreira et al., 2021), but also make students feel satisfied and happy in the learning process. The above effect helps to improve students’ attitudes toward entrepreneurship education, thereby enhancing their learning motivation and actively participating in BSGs (Ranchhod et al., 2014).

The requirement of teamwork ability in BSGs is the key to improving students’ entrepreneurial attitude. Teamwork, task management, and conflict negotiation under the conditions of game simulation can help improve students’ attitudes toward entrepreneurial management projects. In the simulation, different participants face new projects built by new companies, so they are prone to conflicts of opinions. Participants can learn about conflict management of problems through BSGs and improve their negotiation skills to obtain the best solution. Studies have shown that this ability can lead to the changes in entrepreneurial attitude and is the key to fostering students’ entrepreneurial attitude (Arias-Aranda and Bustinza-Sánchez, 2009). Therefore, the use of BSGs as a teaching method to cultivate and enhance students’ entrepreneurial attitude has important reference value.

### Process Perspective of Business Simulation Games

Different from traditional BSGs, in AI-based BSGs, teachers cannot provide offline guidance, but rather serve as supervisors. Moreover, in the teaching process, students use social media to communicate, and the behavior of students is more difficult to observe. Therefore, to study the process of BSGs, it is necessary to consider the appropriate process elements.

Strengthening the research on the development process of BSGs is conducive to in-depth analysis of the connotation, influence mechanism and change process of entrepreneurial attitude. It can improve the teaching methods of BSGs to enhance students’ entrepreneurial attitude. BSGs emphasize that students learn from experience (Costin et al., 2018)and learn while playing games. In the development process, the role of teachers, students’ participation enthusiasm, failure in the game process, enterprise performance and students’ flow experience will affect the development process and the results of BSGs. That will affect students’ entrepreneurial attitude and entrepreneurship educational learning effect. Therefore, we must take these process variables into consideration.

The role of the teacher will to a certain extent affect the attitudes, methods and results of students in the process of participating in the game. In addition to giving students learning opportunities and appropriate guidance, teachers also needs to support and encourage students to improve their learning motivation, thereby enhancing their learning performance. It can be seen that the important connotation and role of the teacher are important variables that we must consider in the development of BSGs. Many scholars mentioned that teachers have diverse roles in BSGs (Robinson et al., 2016). In fact, unlike the traditional teacher role, the role of the teacher in the BSGs is not only the imparter of knowledge, but a variety of roles including supervisors, bystanders, and supporters. This requires teachers to play different roles according to the teaching process, such as acting as a bystander role in the fair competition of multiple teams in the game, and not doing too much interference in the game process. If unfair competition occurs, the teacher needs to act as a supervisor. For teams that fail to operate, teachers need to play the role of supporters. They provide timely guidance, and prompt students to recover from business failures in a timely manner, and so on. Teachers should also become the facilitators of students’ academic work, allowing students to integrate into the classroom environment and be able to independently choose appropriate learning strategies to improve learning motivation and attitudes.

Student participation not only reflects the participant’s own learning state, but also has a certain impact on the learning effect. As a student-centered teaching method, the participation of students in BSGs is particularly important. Learning participation refers to students’ efforts in learning, which shows their commitments and actions to achieve goals (Henrie et al., 2015). Student participation is closely related to teaching methods. Because the change in the form of curriculum participation is also more concerned with mobilizing students’ learning enthusiasm and participation (Hägg and Gabrielsson, 2019). In the course of BSGs, students’ active learning methods are closely related to the transformation of teaching methods. There are three typical teaching modes in higher entrepreneurship education. There are the supply mode of passive education, the demand mode of active participation, the mode that emphasizes the ability of students to use multiple resources to actively solve problems (Nabi et al., 2017). BSGs are a typical representative of the demand model, so BSGs particularly emphasize the active participation of students. On the other hand, the stronger the enthusiasms of students to participate in learning, the more conducive to the development of games, which will promote good teaching effects and improve the quality of entrepreneurial education. Students’ active participation in learning can not only mobilize students to think deeply and memorize learning materials, but also infect and enhance teachers’ teaching enthusiasm, which is conducive to enhancing the entire classroom experience (Auman, 2011). Therefore, students with low learning enthusiasm have a lower degree of participation. Although some scholars have used student participation as a research variable in BSGs, there is no uniform standard for the definition and role of participation, which also brings challenges to research. Therefore, we try to include student participation into the scope of the research variables to study its impact on students’ participation in BSGs.

Failure in the process of simulating the game will help students experience the failure experience of entrepreneurship in advance, and learn to respond better to prepare for future entrepreneurship. This is an important way for students to fully understand the process and risks of business operations and generate entrepreneurial awareness, thereby enhancing their entrepreneurial attitude. In order to study the impact of the failure of business operations on the participants and the final game results in the simulation game, we also included the failure of business operations as a research variable. The failure of business operations has become a common phenomenon in society under the current background that the new crown pneumonia affects economic development (Hwang and Choi, 2021). Similarly, business failure is also a possible situation during the game, so it is necessary to take this situation into consideration. This is also an important manifestation of the authenticity of BSGs. It is a rare learning opportunity that embodies the practical nature of entrepreneurship education (Pérez-Pérez et al., 2021). The failure of entrepreneurship will affect the attitude, confidence and business strategy of students in the process of participation to a certain extent. In the course of the game, if the business fails to simulate the operation, it will face the risk of bankruptcy. This is a simulation of the process of entrepreneurial failure, which helps students to reflect, understand the reasons for bankruptcy, and think about where they made mistakes. Failure makes it impossible for students to continue to improve the follow-up tasks in the BSGs, but it also helps them reflect on the reasons and process of wrong operations, so as to have a deeper understanding and a more complete game experience (Hou, 2015). The failures experienced in entrepreneurship learning also help students learn to manage the negative emotions after failure, and obtain the greatest wisdom of failure on the way of life growth (Pérez-Pérez et al., 2021).

The performance of a simulated enterprise is the learning result that students focus on during the process of participation. It is also an important measure of the success of student teams in entrepreneurship. In order to study the impact of different factors of BSGs on students’ entrepreneurial attitude more comprehensively, we take the performance of simulated companies into consideration. The highest performance is also a reflection of the best performing company, achieving the game goal of defeating other competitors (Peterková and Wozniaková, 2015). Research has also demonstrated that when the performance targets of simulated firms are set too high, participants’ learning may be affected (Stamatelatos and Brooks, 2020). Most scholars evaluate the performance of simulated enterprises from the aspect of financial performance of simulated enterprises. For example, Peterková and Wozniaková (2015) mentioned that the student team will receive relevant financial statement data in each simulated business cycle to compare with other teams. But at the same time he also mentioned the importance of evaluating the individual performance of the team. In addition, some scholars believe that performance should not be a reflection of competitive rankings, but should be students’ attitudes toward courses. The performance of simulated enterprises can effectively test students’ practical entrepreneurial skills, including management ability, decision-making ability, analytical ability, teamwork ability, communication and negotiation ability, etc., which reflect participants’ good control of their own entrepreneurial attitude and ability (Thanasi-Boçe, 2020). Feedback on simulated enterprise performance can also inspire students to think about such issues as the decision-making of enterprise resource allocation, the management of internal teams and the handling of external customer relations in the simulation process. It can be seen that different scholars have different definitions of the evaluation criteria and meanings of simulating enterprise performance. The performance of simulated enterprises will undoubtedly affect the learning attitude of participants and the attitude of real entrepreneurial activities in the future.

Paying attention to the flow experience is to understand the participant’s unconscious concentration on the participation in the simulation game and the impact on the game development and results, which will help to improve the deficiencies in the teaching process of BSGs. Ferreira et al. (2021) mentioned that research on BSGs design focuses more on the experience of participants. In order to study the influence of students’ different flow experiences on participating in BSGs, we included flow experience as a variable in the study. Based on the flow theory (Csikszentmihalyi, 1990), the infectiousness of the game can prompt students to focus on the process and produce a good flow experience, which can increase learning participation and concentration (Yen and Lin, 2020). “Flow” reflects the state where participants are fully focused on something physically and mentally (Csikszentmihalyi, 1975). Studies have pointed out that compared with traditional teaching methods, students are always more likely to experience flow experience in simulation games (Liu et al., 2011). Previous stock knowledge, cooperative mode of the game (Admiraal et al., 2011; Hwang et al., 2011), clarity of game rules, and difficulty in achieving game goals (Wang and Chen, 2010; Hou and Li, 2014) will affect the flow experience of the participants. According to research, participants’ different levels of flow experience will have different effects on their behavioral performance (Hou, 2015) and learning efficiency (Hou and Li, 2014).

## Research Methodology

### Data Collection and Sample Source

In this study, the experimental method was used to conduct research. The experimental group and the control group were set for comparison. The experimental group and the control group measured related variables before and after the experiment, and recorded the specific process of the experimental group. The experiment time is from May 2021 to July 2021. The simulation software used in this experiment is TOP-BOSS, which was used as a competition software for business simulation business competitions. The software trains participants to analyze environmental information, deal with group relationships, and make decisions by simulating the actual business conditions of the enterprise. During the COVID-19 pandemic, the company developing the software has developed BSGs that can be run on mobile phones and tablets to accommodate teaching needs. The experimental group used the BSGs based.

The experimental group is the students participating in the BSGs training course. This course is a compulsory course, which means that students must participate in the course regardless of their original entrepreneurial intentions. Students with different entrepreneurial mentalities participating in the simulation can better observe the role of the course in entrepreneurship education. A difficult problem in the existing research on entrepreneurship education is that the causal relationship is unclear. Do students with high entrepreneurial intentions actively participating in entrepreneurial education, or can entrepreneurial education enhance entrepreneurial intentions? Compulsory courses can reflect the effect of entrepreneurship education on students with different initial states. A total of 105 students in the experimental group participated, and the students voluntarily participated in this survey. The collected results were processed, in which the students who participated in both the pre-test and the post-test were retained. And the unreasonable questionnaire results were eliminated. The data of a total of 83 people were retained.

The control group consisted of business students who did not participate in the simulation training course. The experimental group was in the same school, the same college, and the same grade, with a total of 100 students. The reason is the same school has roughly the same level of intelligence. The same college has basically the same external entrepreneurship support policies, and the influence of external factors can be controlled. The same grade means the education received is roughly the same. The collected results were processed, in which the students who participated in both the pre-test and the post-test were retained. And the unreasonable questionnaire results were eliminated. The data of a total of 83 people were retained.

### Measurement

#### Entrepreneurship Attitude

Entrepreneurship Attitude (EA) draws on the scale of Phan et al. (2002). The items include “Entrepreneurship can accumulate capital and wealth,” “Entrepreneurship can bring oneself a sense of accomplishment,” “Entrepreneurship can enhance one’s social status,” “Entrepreneurship” Can make more contributions to society’ etc. Because this experiment adopts longitudinal research, there are two time points for entrepreneurial attitude measurement. The pre-test is the beginning of the experiment. The post-test is the end of the experiment. EA1 represents the entrepreneurial attitude measured by the pre-test, and EA2 represents the entrepreneurial attitude measured in the post-test, and so on. The pre- and post-test α coefficients of the scale in the control group were 0.817 and 0.863. And the pre- and post-test α coefficients in the experimental group were 0.908 and 0.874, which were both greater than 0.8. The reliability was high.

In order to measure the overall experience of students in the BSGs course, the flow experience (FE) is used for measurement. The scale is referenced from the research of Zhou and Lu (2011), Chang and Zhu (2012), and the Likert scale is adopted. Topic items include “When I participate in BSGs, I often immerse myself in it,” “When I participate in BSGs, I feel that time flies quickly,” “When I participate in BSGs, I feel very happy,” “When I participate in BSGs, I often don’t realize the surrounding environment” and so on. The post-test α coefficients of the scale in the experimental group were 0.821, which was greater than 0.8, and the reliability was ideal.

#### Participation

Due to the large number of participants, it is difficult to judge enthusiasm through observation. If students conduct self-evaluation, they may link their enthusiasm to their grades, and there is a possibility of exaggerating their enthusiasm. Therefore, this research adopts an indirect method. The questionnaire involves multiple variables such as psychological capital, entrepreneurial mentality, entrepreneurial attitude, etc. There are 58 items in total, and the total time to fill out the questionnaire twice is used as a measure of participation enthusiasm. The reason is that if students are willing to spend a longer time filling out the questionnaire to give feedback, it can reflect their participation in the BSGs to some extent.

#### Failure

Failure is measured by a 0-1 variable. 1 indicates that a major failure has been encountered, and 0 indicates no. Students will evaluate whether they have encountered a major failure in BSGs. From the observation results, major failures include a variety of situations, such as the bankruptcy of the operating company, a significant decline in operating performance due to a mistake in decision-making, or the bottom of the industry ranking.

#### Teacher Participation

The teacher participates in the BSGs as a contestant to compete with the student team in the same field, so as to observe whether the student’s mentality changes when there is a relatively experienced competitor in the industry. Teacher participation is measured using dummy variables. 1 means that the teacher participates in the game as a contestant and 0 means no.

#### Operating Performance

If students make multiple rounds of decision-making in BSGs, the final score ranking is used as the operating performance cannot reflect the process of the competition, which refer to Newbery et al. (2018) for the performance measurement method. This article defines operating performance. It is the number of times the profit is positive in the business process. A total of 16 decisions were made in this experiment, with the maximum value being 16 and the minimum value being 0.

### Data Processing

The data analysis and processing of this study includes two aspects. One is to conduct a paired sample *T*-test on the pre-test and post-test. Entrepreneurial attitude of the control group and the experimental group are observed whether there is any change. The second is to use fsQCA fuzzy set qualitative comparative analysis to explore entrepreneurship. How do the antecedents and conditions of attitude improvement interact? Qualitative comparative analysis (QCA) has been widely used in recent years. Based on the principle of Boolean algebra, it can analyze different combinations of conditional variables and reveal complex causal relationships. The causal variables in this study include both categorical and continuous variables, so the fuzzy set qualitative comparison method (fsQCA) is used. In the variable calibration, the 95%, 50%, and 5% (Fiss, 2011) quantile values of the three variables of EA, PA, and FE are used as the threshold values of complete membership, intersection point, and complete non-membership. In the configuration analysis, the consistency threshold is set to 0.8, the case coverage threshold is 1, and the PRI value is 0.75.

## Results

### Descriptive Statistics

Table 1 is descriptive statistics. The average value of EA2 in the control group is 22.49, which is higher than EA1, and the average value of EA2 in the experimental group is also higher than EA1, indicating that the average level of entrepreneurial attitude has increased at the two time points. Whether there is a significant change in entrepreneurial attitude still needs further verification.

**TABLE 1:** Descriptive statistics.

	Control group				Experimental group			
	Max	Min	Mean	*s.d.*	Max	Min	Mean	*s.d.*
EA1	28	14	22.04	3.74	28	13	22.30	3.95
EA2	28	10	22.49	3.52	28	16	23.17	3.15
PA	−	−	−	−	3948	94	662.26	80.18
FE	−	−	−	−	28	14	22.43	0.38
FA	−	−	−	−	1	0	0.47	0.05
PER	−	−	−	−	12	0	6.40	0.39
TA	−	−	−	−	1	0	0.74	0.44

### Paired Sample *T*-Test

A paired-sample *T*-test was used for the main variables of the control group and the experimental group. The results in Table 2 showed that from the results of the control group, although the mean value of EA2 was 0.468 higher than that of EA1, the difference in the mean value was not significant from the results of the *T*-test. It shows that for the control group, the entrepreneurial attitude of the pre-test and post-test did not change significantly. For the experimental group, the mean value of EA2 is 0.872 higher than that of EA1, which is significant at the level of 0.01. The experimental group’s entrepreneurial attitude has undergone a significant change.

**TABLE 2:** Paired sample *T*-test.

Paired sample	Control group			Experimental group		
	Mean	*s.d.*	*t*	Mean	*s.d.*	*t*
EA2 - EA1	0.468	4.520	1.092	0.872**	3.009	2.688

**P < 0.01.

### Univariate Necessity Analysis

A necessary analysis of a single variable can determine whether the condition is a necessary condition for the result. According to Ragin (2010), when the consistency level is higher than 0.9, this condition is a necessary condition for the result. The results in Table 3 show that the consistency of all the conditional variables is less than 0.9, indicating that the necessary conditions do not exist. It is indicating that the improvement of entrepreneurial attitude cannot be explained by a separate condition, that is, the improvement of entrepreneurial attitude is not caused by What is determined by a single fixed variable is the result of multiple factors. Therefore, it is necessary to configure and analyze the condition variables to find out the combination of multiple conditions.

**TABLE 3:** Analysis of the necessary conditions.

Antecedent condition	Consistency	Coverage
FA	0.436	0.492
∼FA	0.564	0.554
TA	0.782	0.553
∼TA	0.218	0.447
PA	0.600	0.712
∼PA	0.716	0.676
FE	0.613	0.665
∼FE	0.654	0.666
PER	0.592	0.673
∼PER	0.670	0.655

### Sufficiency Analysis of Conditional Configuration

Configuration analysis can reveal the adequacy of the results caused by multiple configurations. Fuzzy set analysis can obtain three solutions: complex connection, simple solution and intermediate solution. The intermediate solution is the most commonly used result in fsQCA, which is close to the theoretical reality without being too complicated. Combining simple solutions can determine the core conditions of each condition combination and marginal conditions. According to Ragin’s suggestion, the consistency threshold is set to 0.8, the case coverage threshold is set to 1, and the PRI value is set to 0.75. The results in Table 4 show that the agreement between the single solution and the overall solution is greater than 0.8, which is higher than the 0.75 suggested by Ragin, and the result is highly effective. The coverage of the overall solution is 0.390, which is relatively low, indicating that there may be other conditions that have not been considered in this study.

**TABLE 4:** Conditional configuration.

Antecedent condition	M1	M2	M3	M4
FA	⊗	⊗	⊗	🌑
TA	🌑	🌑	⊗	⊗
PA		🌑	⊗	🌑
FE	⊗	🌑	🌑	⊗
PER	🌑		⊗	⊗
Raw coverage	0.222	0.218	0.043	0.041
unique coverage	0.089	0.084	0.043	0.041
consistency	0.883	0.897	0.865	0.882
Solution coverage	0.390			
Solution consistency	0.865			

*🌑, core condition exists; ⊗, core condition is absent; 🌑, edge conditions exist; ⊗, edge conditions is absent.*

### Robustness Test

In this study, the consistency threshold was adjusted from 0.8 to 0.85, and the variable cross-cutting value was adjusted between -25% and 25%. The configuration quantity, configuration form, consistency and coverage rate did not change substantially, so the above research conclusions are robust.

## Discussion

### The Formation Model of Improving Students’ Entrepreneurial Attitude

**FIGURE 1:**
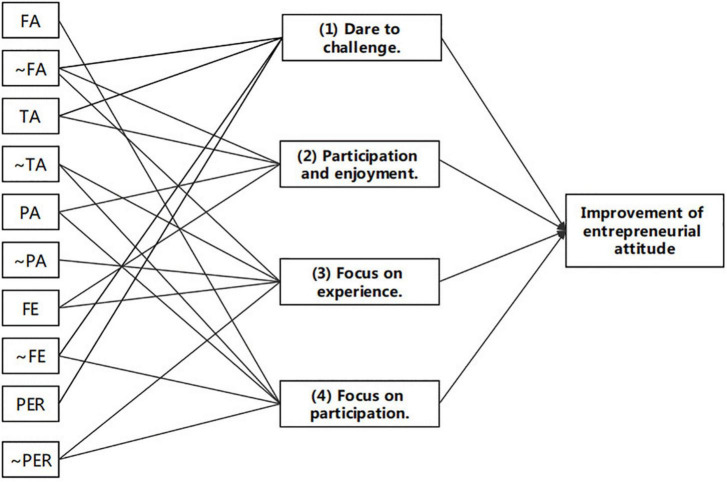
Formation mode of entrepreneurship attitude improvement.

Based on the above results, we have identified five modes for mobile-based simulation games to enhance college students’ entrepreneurial attitude, which are shown in Figure 1.

(1) Dare to challenge. Configuration 1 shows that when the teacher participates in the competition, the student’s team has good business performance and no major failures. Even if the course’s flow experience is not good, it can still effectively improve the entrepreneurial attitude. Teachers with relatively rich experience participated in the competition as participants. Some students showed a sense of competition and tried to fight against teachers in competition. According to the observation results during the competition, some student teams attach great importance to the division of labor and cooperation, so the atmosphere of cooperation is strong. They will adopt a variety of strategies to make decisions, such as imitating the teacher’s business strategy to make decisions, analyzing the strategies of competitors, and even adopting other strategies. The student team conducts an alliance against the company run by the faculty to prove the confrontation. Judging from the results of the competition, these students often rank in the forefront of competition results. This attitude of being strong when it comes to strength can effectively enhance the entrepreneurial attitude.

(2) Participation and enjoyment. Configuration 2 shows that when teachers participate in the competition, students actively participate without major failures, have a better experience of the course flow, and can improve their entrepreneurial attitude. This part of students pays special attention to participation in the competition process. Teacher participation can bring a different experience, and their own participation is high. The course experience is good. In addition, they have not encountered major failures in the business process, which may give students a kind of business operation. The feeling of relative smoothness can also improve the entrepreneurial attitude to a certain extent.

(3) Focus on experience. Configuration 3 shows that when the teacher does not participate and the students’ enthusiasm participation is not high, if the students do not experience failure, but the curriculum flow experience is better. The entrepreneurial attitude can still be effectively improved. This reflects the importance of flow experience in BSGs courses. Even if other factors are not particularly ideal, flow experience has a positive effect on improving self-efficacy. This inspired the need to pay attention to the role of flow experience in the curriculum design of BSGs. In the course design, factors such as how to match skills with challenges and ensuring the playability of the game all need to be considered. During the operation of the game, how to let students set clear goals and concentrate attention also need to be paid attention to. After the course is over, it is necessary to summarize and reflect on the effects and consequences of the flow experience, and to be able to accumulate experience in the process of repeated cycles.

(4) Focus on participation. Configuration 4 shows that when the teacher does not participate in the competition, the experience of the course flow experience is poor and the performance is not ideal, but the students actively participate. Even if they encounter a major failure, the entrepreneurial attitude is still improved. In this state, students have put in more effort, but they still encounter failures and poor business performance. This part of the students may have a “do their best” mentality, not focusing on grades and course experience, but caring about whether they work hard to participate.

### Possible Uses of Artificial Intelligence in Business Simulation Games

According to the above analysis, we found the possible application ways of AI in BSGs, specifically including the following ways.

Artificial intelligence participates in BSGs in various capacities such as mentors or players (Bikovska et al., 2006). AI can mimic human behavior in the game (Pavlicek et al., 2014). Depending on teaching needs, AI may need to simulate participants at different levels, for example, creating comparable competitors and game modes for participants of different skill and experience levels (Dreżewski and Solawa, 2021). In the existing BSGs, there are no qualified computer players. The reason is that the strategies of BSGs are diversified, and it is difficult for existing software to respond accordingly according to the strategies of human players. AI can make corresponding strategies according to different algorithms through machine learning. The way the AI plays the player is beneficial. For example, AI can act as a guide for game novice players, guiding players to master basic game strategies. This includes not only an explanation of the game’s content, form, method, etc., but also guidance on the deficiencies of the current participants in the simulation process (Bikovska et al., 2006). AI can also act as a strong competitor, thereby simulating the situation that a company may encounter a strong competitor in reality. The simulation method is undoubtedly beneficial to the exercise of participants’ psychological quality and the improvement of strategic decision-making skills (Fuchsberger, 2016).

Artificial intelligence can provide targeted feedback on business failures. Learning from failure is an important part of entrepreneurial learning. In BSGs, it is inevitable that participants will fail in their operations. This requires participants to reflect, and thus to learn from failure. Existing teaching models rely on teachers’ guidance to repair failures. However, due to the limited ability and time of teachers, it is difficult to provide detailed guidance and analysis. If AI can analyze the failures encountered and provide targeted feedback, it will be very beneficial to repair the confidence of the participants. For example, the automated supply chain of AI can predict possible future supply chain disruptions and give early warnings through the operations of participants, so as to remind risks and intervene in time (Brauner et al., 2019). AI can find suitable operation paths for players in the game world, so as to maximize the effectiveness of the player character and smoothly pass through all obstacles (Dreżewski and Solawa, 2021). Through this learning method, participants can truly learn from failure, accumulate experience, and build confidence for the next venture.

Artificial intelligence can also start from improving the flow experience and enhance the entrepreneurial attitude of participants. The flow experience of the BSGs is very important. Even if some participants are not good in all aspects, but the flow experience is good, they can still improve their entrepreneurial attitude. AI will provide unique and compelling experiences based on the situation of different participants, not limited to the goal of seeking maximum success for participants (Dreżewski and Solawa, 2021). What’s more, AI can also detect the participants’ breathing rate and other physical data to understand their adaptability to the game process, so as to achieve the goal of maximizing the physical and mental satisfaction of the participants (Xi, 2020). AI can provide the development and transformation of different scenarios according to the different characteristics of participants through the initial definition of conditions and continuous identification of the operating behavior of the system, which is conducive to the participants’ immersive experience and promoting the flow experience (Bikovska et al., 2006). Furthermore, Players have a variety of behaviors and reactions during the game, such as cheering in the process of turning losses into profits, applauding when the rankings are announced, and so on. If AI can collect this information, it will be able to determine which aspects of the participant experience the best, so as to provide feedback for the design of the game. For example, AI can change the emotional state of participants by adjusting goals or paths (Dreżewski and Solawa, 2021), which is beneficial for participant flow experience and the improvement of entrepreneurial attitude.

Artificial intelligence can also provide timely feedback on business performance. In the process of BSGs, business performance is an indicator that participants value very much. Due to the short decision-making time and the large number of participating teams, it is difficult for teachers to provide targeted guidance for each company. It is very important for participants to learn how to analyze business decisions. If the teacher fails to answer the confusion in the business process in a timely manner, the participants may lose interest. These problems can be avoided if AI can provide feedback when participants need it. Timely feedback is beneficial for improving student engagement and achievement. Research has shown that AI can use data to understand feedback students’ growth dynamics (Ryoo and Winkelmann, 2021). Participants are able to gain a continuous decision-making experience through AI, and understand the impact of the decision on present and future situations through immediate performance to make better responses in a timely manner (Bikovska et al., 2006). AI can also set up repetitive simulations, giving participants the opportunity to re-engage in decision-making, which is consistent with real-world business environments (Bikovska et al., 2006). The logic analysis and decision optimization of AI are the functions that students think have the most development potential and the highest utilization (Fuchsberger, 2016). In addition, there are real-time follow-up and feedback requirements such as path-finding, analytical decision-making, and teamwork (Dreżewski and Solawa, 2021).

Artificial intelligence plays a role in improving student engagement. The fun of the game, the stability of the technology, the simulation degree of simulation and other factors all have an impact on the degree of participation of the students. Enhancing student engagement can start from different solutions such as the game project itself, the problems in the simulation process, and the way of teaching behavior (Larraza-Mendiluze et al., 2020). Studies have shown that participants can improve self-efficacy and enhance trust in technology when interacting with AI technology (Brauner et al., 2019). Human-machine cooperation also includes not only the vertical process of strategic analysis and decision-making, but also the horizontal aspects of data collection and analysis (Yang et al., 2021). In order to achieve the best functional effect, AI systems are also continuously improved internally as external actors and other environments change (Yang et al., 2021). During the game, AI can collect relevant data on the factors affecting student participation, and integrate this data to provide game designers with suggestions for improvement, which can promote the improvement of simulation games and the improvement of student participation.

### Limitation

The limitation of this article is that when using fuzzy set qualitative comparative analysis to discuss the teaching effect of mobile BSGs, the coverage of the overall solution is low, indicating that there are other factors that have not been considered in this study. The teaching process of BSGs covers a variety of factors, and future research can continue to explore the role of other factors.

Another major flaw is that several application directions of AI in BSGs proposed in this study may be technically difficult to achieve. This study considers how AI and BSGs can be combined based on the ways in which existing BSGs enhance entrepreneurial attitude. AI has not really been involved in BSGs, which may present technical difficulties. In addition, the effect of AI’s real involvement in BSGs remains to be verified.

## Conclusion

This article explores how AI can be combined with BSGs. Given that little research has been done on this topic, we analyzed the process of BSGs to see where AI could be applied. A quasi-experiment was employed to explore how BSGs changed participants’ entrepreneurial attitude. It was found that BSGs can significantly improve entrepreneurial attitude. This change can be carried out through four configurations consisting of five antecedent conditions.

Specifically, it includes daring to challenge, participation and enjoyment, focus on experience and focus on participation. The combination of these four configurations shows us that whether students experience major failures in participating in BSGs, receive teacher participation assistance, have active participation, have better flow experience, and good business performance, and will affect students’ entrepreneurial attitude in varying degrees. This affects the effectiveness of entrepreneurship education. Based on this, we believe that AI can improve the effect of BSGs by playing different roles, providing failure feedback, improving game experience, analyzing business performance, etc., so as to better play an effect of AI in entrepreneurship education. The contribution of this paper is to emphasize the possibility of combining AI and BSGs, and to point out several ways in which AI can intervene in BSGs, providing reference for the improvement of BSGs.

## Data Availability Statement

The raw data supporting the conclusions of this article will be made available by the authors, without undue reservation.

## Author Contributions

JC contributed to the work concept or design and data collection. YC, JC, and RO drafted the manuscript. JW and QC make an important revisions to the manuscript. All authors approved the final version of the manuscript for publication.

## Conflict of Interest

The authors declare that the research was conducted in the absence of any commercial or financial relationships that could be construed as a potential conflict of interest.

## Publisher’s Note

All claims expressed in this article are solely those of the authors and do not necessarily represent those of their affiliated organizations, or those of the publisher, the editors and the reviewers. Any product that may be evaluated in this article, or claim that may be made by its manufacturer, is not guaranteed or endorsed by the publisher.
