# Erratum: How to Use Artificial Intelligence to Improve Entrepreneurial Attitude in Business Simulation Games: Implications From a Quasi-Experiment

**DOI:** 10.3389/fpsyg.2022.915921

**Published:** 2022-04-26

**Authors:** Frontiers Production Office

**Affiliations:** Frontiers Media SA, Lausanne, Switzerland

**Keywords:** business simulation game, artificial intelligence, entrepreneurial attitude, quasi-experiment, fuzzy set qualitative comparative analysis, process perspective

Due to a production error, the article was published in the wrong journal. It is now published in the correct journal, Frontiers in Psychology. The publisher apologizes for this mistake.

The original version of this article has been updated.

## Publisher's Note

All claims expressed in this article are solely those of the authors and do not necessarily represent those of their affiliated organizations, or those of the publisher, the editors and the reviewers. Any product that may be evaluated in this article, or claim that may be made by its manufacturer, is not guaranteed or endorsed by the publisher.

